# Brain Volume Loss After Stereotactic Laser Interstitial Thermal Therapy in Patients With Temporal Lobe Epilepsy

**DOI:** 10.1111/jon.70039

**Published:** 2025-04-08

**Authors:** Sebastian Johannes Müller, Eya Khadhraoui, Olga Kukhlenko, Johannes Schwarzer, Jürgen Voges, I. Erol Sandalcioglu, Daniel Behme, Friedhelm Schmitt, Lars Büntjen

**Affiliations:** ^1^ Clinic for Neuroradiology Otto‐Von‐Guericke‐University Magdeburg Magdeburg Germany; ^2^ Clinic for Neurology Otto‐Von‐Guericke‐University Magdeburg Magdeburg Germany; ^3^ Clinic for Stereotactic Neurosurgery Otto‐Von‐Guericke‐University Magdeburg Magdeburg Germany; ^4^ Clinic for Neurosurgery Otto‐Von‐Guericke‐University Magdeburg Magdeburg Germany; ^5^ Stimulate Research Campus Magdeburg Germany

**Keywords:** epilepsy, FastSurfer, hippocampal region, laser interstitial thermal therapy, volumetry

## Abstract

**Background and Purpose:**

Temporal lobe epilepsy is the most common form of focal epilepsy. MR‐guided laser interstitial thermal therapy (LITT) of the amygdalohippocampal complex has become an established therapy option in case of drug resistance. Long‐term anatomic network effects on the brain due to deafferentiation have not yet been evaluated.

**Methods:**

We analyzed brain volumes of 11 patients with temporal lobe epilepsy before and 1‐year after hippocampal LITT with FastSurfer segmenting T1‐weighted data. Additionally, we performed visual ratings and measurements.

**Results:**

A total of 11 patients with temporal lobe epilepsy (7 left‐sided, 4 right‐sided) were included (5 females); the mean age years (±standard deviation) at surgery was 41.5 (±18.4) years. The mean postoperative defect size was 1427 (±517) mm^3^. Volumetry as well as visual ratings found a progressive volume loss after left‐sided surgery in the ipsilateral temporal lobe, the contralateral (right) part of the thalamus, and especially contralateral (right) fusiform cortex. These changes could not be detected for right‐sided surgery.

**Conclusion:**

A (partial) ablation of the left (dominant) hippocampus appears to exert long‐term effects on the right thalamus and right‐sided temporal cortices. However, we could not observe this effect in the reverse direction. Volumetric studies for larger cohorts should be conducted to investigate these findings.

## 1 Introduction

Heterogeneous brain volume loss has been observed following stroke [1] or traumatic brain injury [2]. We hypothesize that atrophy may also occur after neurosurgical procedures. Epilepsy surgery, with its standardized procedures, provides an excellent field for studying and identifying specific atrophy patterns. Current developments revealed the network character of epilepsy [3, 4]. Particularly, the hippocampus represents a significant junction and is connected to many structures [5].

Anterior temporal lobectomy is the gold standard in the treatment of (drug resistant) temporal lobe epilepsies (TLE) with success rates of 60%–80% regarding seizure freedom [6]. Less radical surgical procedures, such as selective amygdalo‐hippocampectomy [7], are often better accepted by patients.

**FIGURE 1:**
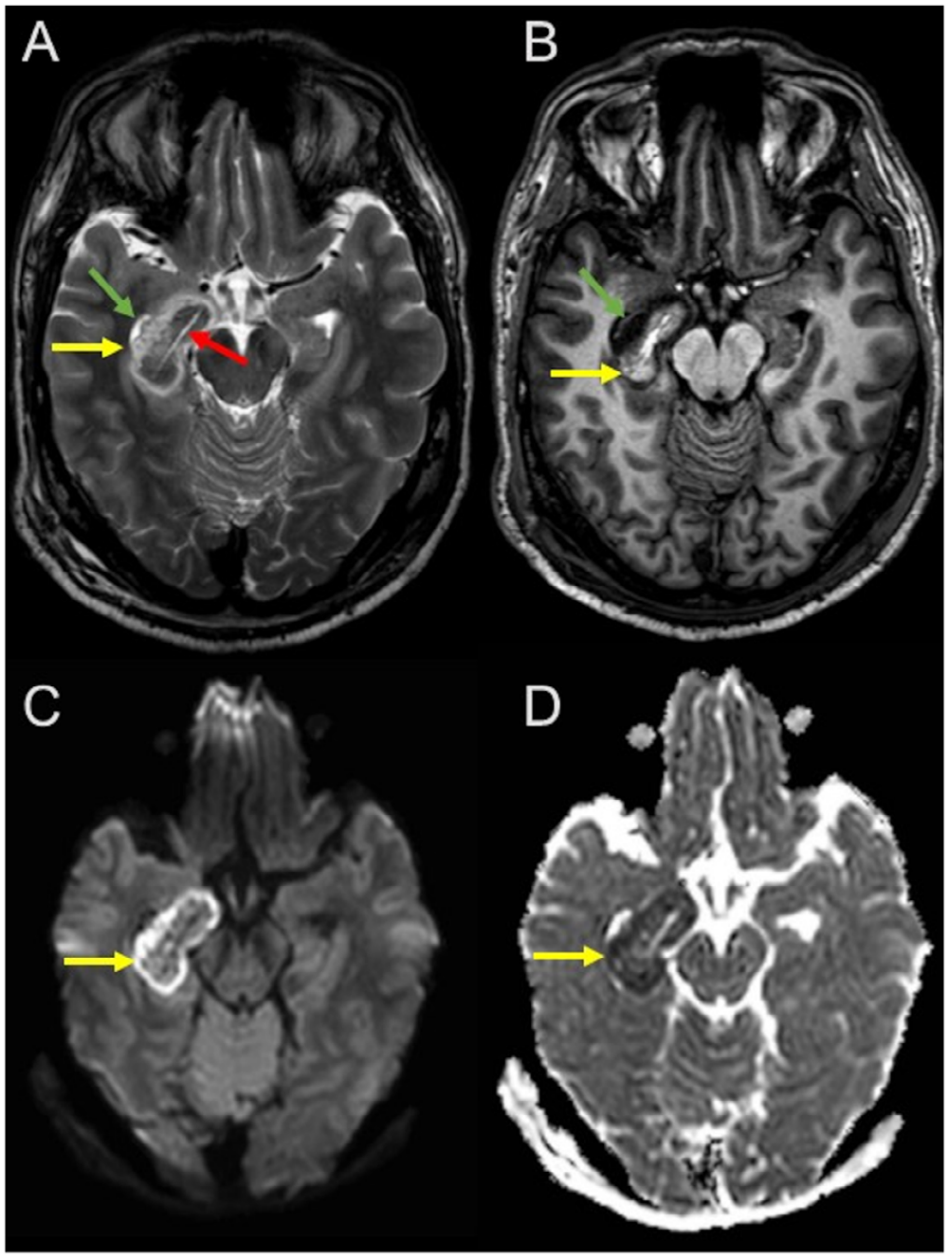
Introduction example. Early postoperative MRI following laser interstitial thermal therapy in a patient with right‐sided temporal lobe epilepsy. In the right amygdala and hippocampal region beside the temporal horn (green arrow), a central elongated early postoperative defect is visible following laser ablation (red arrow) surrounded by thermal edema (yellow arrows) that closely resembles infarction‐related edema due to diffusion restriction. (A) Transversal T2‐weighted Turbo‐Spin‐Echo; (B) transversal T1‐weighted Turbo‐Field‐Echo; bottom: transversal diffusion‐weighted imaging; (C) B1000; (D) apparent diffusion coefficient map.

However, automated brain volumetry following brain surgeries is also particularly challenging due to cortical abnormalities [8]. Such defects are significantly smaller with minimally invasive procedures, for example, the MR‐guided laser interstitial thermal therapy (LITT) [9]. Therefore, automated volumetry should be more feasible after this approach compared to invasive methods. Figure 1 demonstrates an MRI following LITT.

Therefore, the goal of this MR study is to investigate the long‐term effects of this minimally invasive therapy on the cerebral cortex and brain tissue volumes.

To do this, we performed automated segmentation of the T1‐weighted MRI sequences before and 1 year after surgery using FastSurfer [10, 11]. In addition, we conducted a manual volumetric analysis of the lesioned brain tissue.

## 2 Methods

### 2.1 Study Design

This retrospective study was ethically approved by the institutional review board and adhered to the 2013 Declaration of Helsinki. The institutional review board waived the requirement for informed consent because of the retrospective nature of the study. All methods were performed in accordance with relevant guidelines and regulations.

### 2.2 Participant Population

We screened our picture archiving and communication system (PACS) for patients with TLE and LITT between 01/01/2018 and 12/31/2023. Inclusion criteria were TLE, an MR‐guided LITT with pre‐ and postoperative MRI. Exclusion criteria were the non‐existence of a three‐dimensional (3D) T1‐weighted sequence in the 1‐year follow‐up MRI scans. Due to postoperative changes and edema, 3‐ and 6‐month‐follow‐up MRIs were not included.

One year follow‐up data were available in the International League Against Epilepsy (ILAE) classification [12].

### 2.3 MRI Protocols and Scanners

3D T1‐Datasets with a voxel‐size of 1 mm × 1 mm × 1 mm were performed on 3T PRISMA (Siemens Healthineers AG, Munich, Germany; 3D‐T1‐Magnetization Prepared—RApid Gradient Echo; repetition time [TR] 2300 ms; echo time [TE] 3 ms) and 3T Achieva (Philips Nederland B.V., Eindhoven, the Netherlands; 3D‐T1‐weighted‐turbo field echo, TR 10 ms; TE 5 ms).

### 2.4 Volumetry

We ran FastSurfer (Version 2.3.0, German Center for Neurodegenerative Diseases DZNE, Bonn, Germany, https://deep‐mi.org/research/fastsurfer) on T1‐weighted data of MRI and calculated brain volumes according to the Desikan–Killiany–Tourville (DKT) [13] atlas. Additionally, we performed a parcellation based on Yeo's 17‐network resting‐state map [14]. For this purpose, the FreeSurfer (Version Stable v7.4.0, Laboratory for Computational Neuroimaging at the Martinos Center for Biomedical Imaging, Massachusetts General Hospital, Harvard Medical School, Boston, MA, USA, https://surfer.nmr.mgh.harvard.edu) procedures mri_surf2surf [15] and mris_anatomical_stats were applied to the FastSurfer results. A manual masking of the lesions or defect areas was not performed.

We also tried to run the additional HypVINN script [16], when possible, to assess the anterior commissure and the fornix.

**FIGURE 2:**
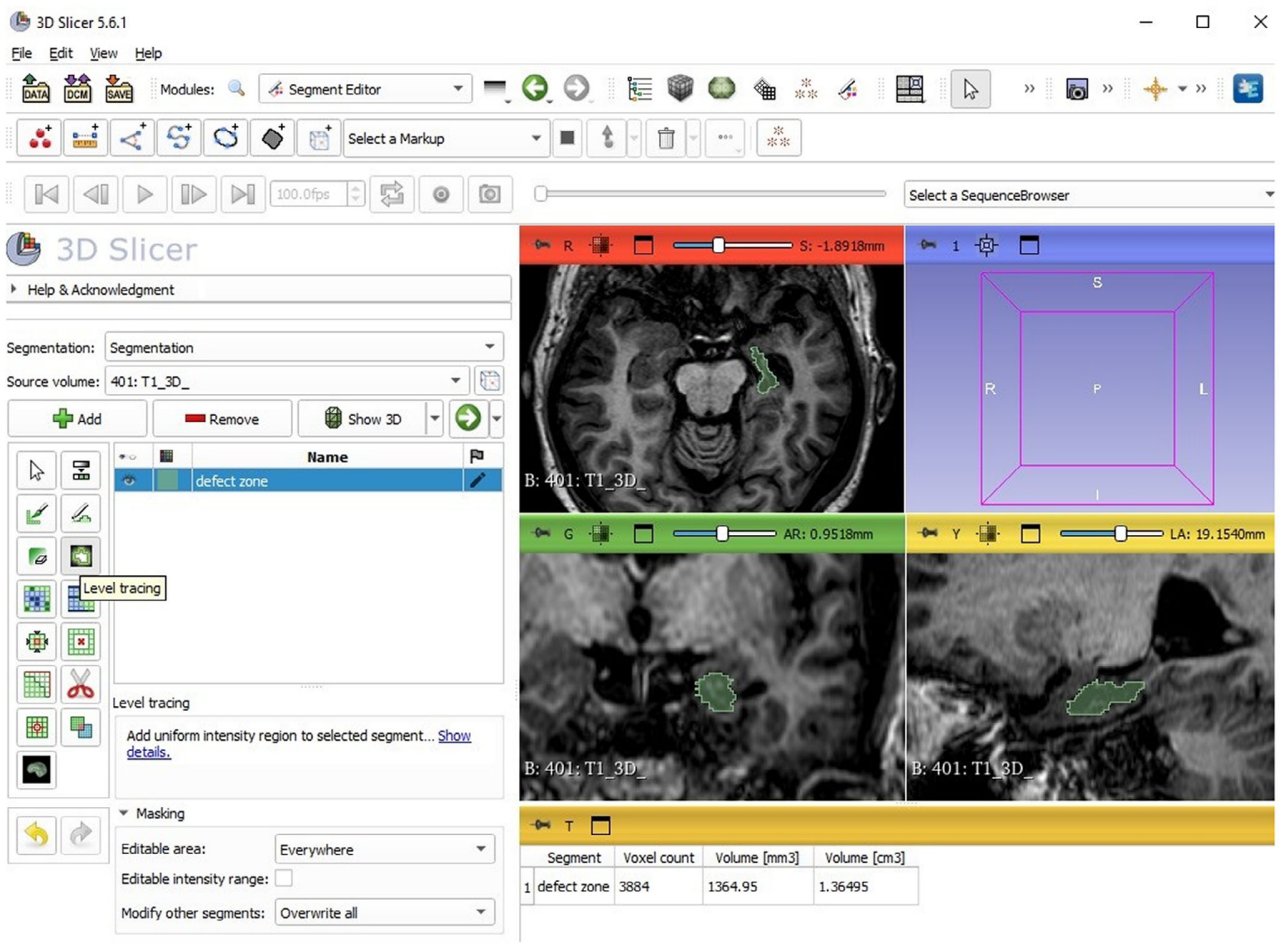
3D Slicer example demonstrates a manual volumetric measurement of the defect zone (green) following laser interstitial thermal therapy. 3D, three‐dimensional; AR, anterior right; B, brain series number and name; cm^3^, cubic centimeter; I, inferior; L, left; LA, lateral anterior; mm^3^, cubic millimeter; P, posterior; R, right; S, superior.

We compared pre‐surgery and 1‐year follow‐up and measured the T1‐weighted defect zone with 3D Slicer [17] (Version 5.6.1, Brigham and Women's Hospital, Boston, USA, https://www.slicer.org) in mm^3^, that is, cerebrospinal fluid (CSF)‐isointense defect on T1‐weighted sequences. We carefully subtracted the ventricle system in cases of possible conflicts (via comparison with the pre‐surgery images). Figure 2 shows an example.

Two independent neuroradiologists visually rated the entorhinal cortex atrophy (ERICA) score [18] (0–3) and Schelten's medio temporal atrophy (MTA) score [19] (0–4) before and 1‐year after surgery. The ERICA score measures atrophy of the entorhinal cortex and parahippocampal cortex and is visually assessed in coronal slices at the level of the mammillary bodies. The MTA score captures the width of the choroid fissure, the width of the temporal horn of the lateral ventricle, as well as the height of the hippocampus.

We performed a functional (language) MRI for each patient to determine the dominant hemisphere. As the effects of valproate on volumetry [20, 21, 22] have been extensively described, we conducted an additional analysis of the antiepileptic medication administered before and after LITT.

### 2.5 Statistical Analysis

We used Python3 (Version 3.9.18, Python Software Foundation, Wilmington, USA, https://www.python.org) for statistical programming and histogram/image construction. As a baseline analysis, we compared the DKT volumes between the initial and follow‐up MRIs. Significance level *p* was set to 0.05. Additionally, we used the false‐discovery‐rate (FDR) of Benjamini and Hochberg [23] and Bonferroni's correction [24]; hence, we tested 100 variables (50 for each hemisphere), and we set *p* (Bonferroni corrected) = 0.05/50 = 0.001. We used multiple paired *t*‐test to compare pre‐ and post‐surgery brain volumes. To test interrater reliability, we calculated intraclass correlation coefficient (ICC) (c, 2) and estimated the 95% confidence intervals with R (Vienna, Austria, https://www.r‐project.org/) (packages irr, readxl, lpSolve, and psych). The value was interpreted following Koo and Li [25]. Pearson's correlation was used to assess the association between size of defect and seizure freedom.

## 3 Results

### 3.1 Participants

**FIGURE 3:**
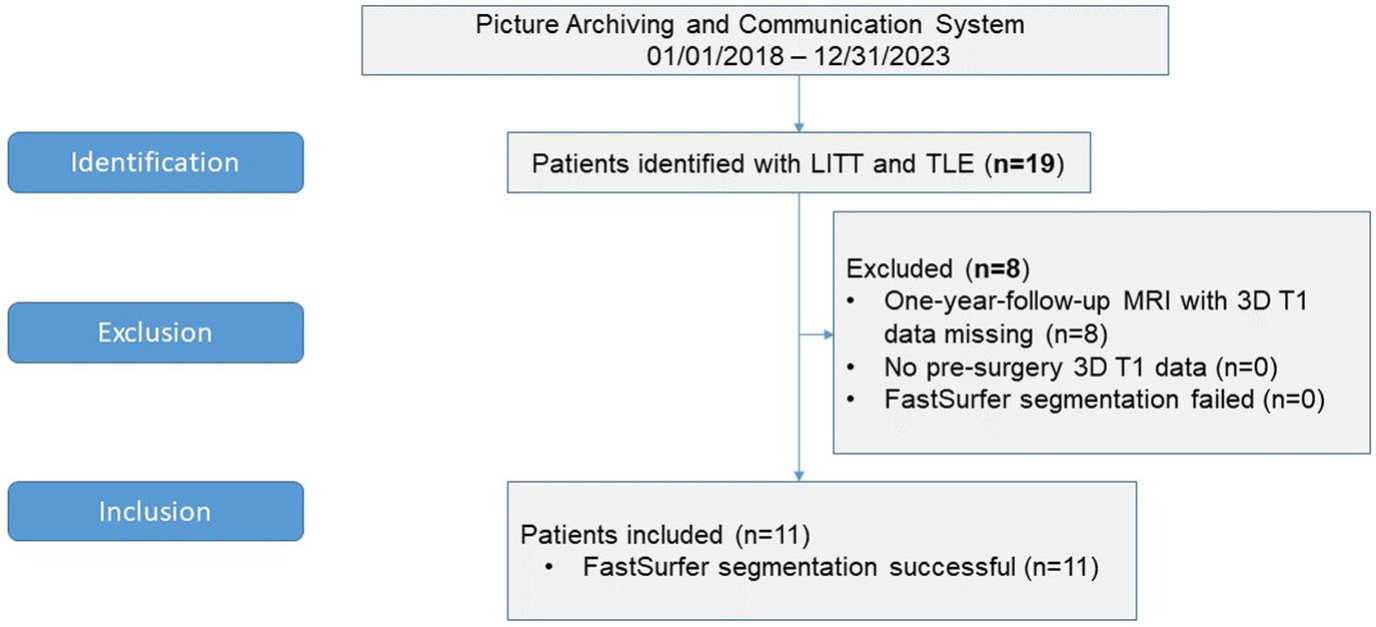
Inclusion Flow Chart. 3D, three‐dimensional; LITT, laser interstitial thermal therapy; *n*, number of patients; TLE, temporal lobe epilepsy.

We found 19 patients in our PACS who met the criteria. Eight patients had to be excluded because of missing/not adequate follow‐up MR scans. Figure 3 shows the inclusion flow chart.

The mean age of the finally included 11 patients with TLE was 41.5 ± 18.4 years (range 17–69 years; 5 females) at the time of LITT. The minimal‐invasive amygdalo‐hippocampal ablation was performed seven times left‐sided and four times right‐sided. Mean epileptic onset age was 16 ± 8 years.

### 3.2 Basic Results

Mean interval between surgery and evaluated follow‐up MRI was 15 ± 6 (range 8.8–26.1) months. Mean outcome (ILAE) was 2.5 ± 1.8 (Median 1; 6 out of 11 patients had an ILAE score of 1, 1 year after LITT).

Table 1 summarizes the important mean volumes and standard deviations of the FastSurfer volumetry using the DKT atlas.

**TABLE 1:** Mean volume ± standard deviation (mm^3^) of relevant structures of the Desikan–Killiany–Tourville‐Atlas and significance *p* without correction.

Brain region	All patients (*n* = 11)			Left‐sided TLE (*n* = 7)			Right‐sided TLE (*n* = 4)			Dominant side LITT (left, *n* = 5)		
	Volume pre‐surgery MRI in mm^3^	Volume 1‐year follow‐up MRI in mm^3^	*p*	Volume pre‐surgery MRI in mm^3^	Volume 1‐year follow‐up MRI in mm^3^	*p*	Volume pre‐surgery MRI in mm^3^	Volume 1‐year follow‐up MRI in mm^3^	*p*	Volume pre‐surgery MRI in mm^3^	Volume 1‐year follow‐up MRI in mm^3^	*p*
Left‐thalamus	7159	6789	0.19	7023	6329	0.0004	7396	7595	0.78	7317	6536	0.002^a^
Left‐hippocampus	3540	2551	0.004	3294	1731	0.0001^*^	3971	3986	0.96	3349	1665	0.00002^*^
Left‐amygdala	1676	1093	0.003	1625	741	0.0003^*^	1765	1710	0.59	1718	731	0.002^a^
Right‐thalamus	7095	6754	0.06	7161	6740	0.001^*^	6978	6779	0.68	7426	6926	0.003^a^
Right‐hippocampus	4052	3252	0.008	4231	3840	0.008^a^	3740	2223	0.05	4196	3724	0.008^a^
Right‐amygdala	1841	1362	0.02	1777	1656	0.04	1954	848	0.03	1784	1650	0.08
ctx‐lh‐entorhinal	1565	1156	0.01	1548	888	0.0006^*^	1596	1624	0.88	1589	941	0.008^a^
ctx‐lh‐fusiform	6815	6151	0.08	6958	5691	0.002^a^	6564	6955	0.55	7270	5854	0.01^a^
ctx‐lh‐parahippocampal	1889	1642	0.04	1881	1433	0.001^*^	1902	2009	0.32	1980	1513	0.006^a^
ctx‐rh‐entorhinal	1485	1124	0.0001^*^	1491	1145	0.002^a^	1474	1087	0.02	1487	1117	0.006^a^
ctx‐rh‐fusiform	7354	6663	0.003	7369	6449	0.001^*^	7326	7039	0.48	7490	6443	0.006^a^
ctx‐rh‐parahippocampal	1798	1644	0.03	1825	1619	0.02^a^	1752	1687	0.55	1850	1587	0.02

Abbreviations: ctx, cortex; lh, left hemisphere; LITT, laser interstitial thermal therapy; mm^3^, cubic millimeter; *n*, number of patients; *p*, probability (significance) value; rh, right hemisphere; TLE, temporal lobe epilepsy.

^a^Significant using the Benjamini–Hochberg false‐discovery‐rate.

^*^Significant using the Benjamini–Hochberg false‐discovery‐rate and the Bonferroni corrected significance level of 0.1%.

The corrected significance level of 0.001 was reached by the ablated hippocampus and amygdala after left‐sided LITT, as well as the left and right parts of the thalamus and the fusiform cortex of both hemispheres.

The use of the Benjamini–Hochberg FDR instead of the Bonferroni correction led to more frontal and temporal structures of both hemispheres being recognized as having a significantly reduced volume.

In the 1‐year follow‐up after a left‐hemispheric operation, we also observed an increase in the volume of the right temporal horn, along with a reduction in the entorhinal gyrus and the medial and lateral orbitofrontal cortices. The segmented brain volumes with the highest differences are shown in Table 2.

**TABLE 2:** Differences in percent (pre‐surgery vs. 1‐year follow‐up MRI) for areas with an increase/decrease >15% (deviations up to 10% could be explained to inter‐scanner deviations).

	All patients (%)	Left‐sided LITT (%)	Right‐sided LITT (%)
Left‐hippocampus	−27.93	−47.44	0.38
Right‐hippocampus	−19.75	−9.24	−40.55
Left‐amygdala	−34.78	−54.43	−3.11
Right‐amygdala	−26.03	−6.82	−56.60
ctx‐lh‐entorhinal	−26.18	−42.65	1.77
ctx‐rh‐entorhinal	−24.35	−23.25	−26.30
ctx‐lh‐fusiform	−9.74	−18.21	5.96
ctx‐rh‐fusiform	−9.39	−12.50	−3.92

Abbreviations: ctx, cortex; lh, left hemisphere; LITT, laser interstitial thermal therapy; rh, right hemisphere.

**FIGURE 4:**
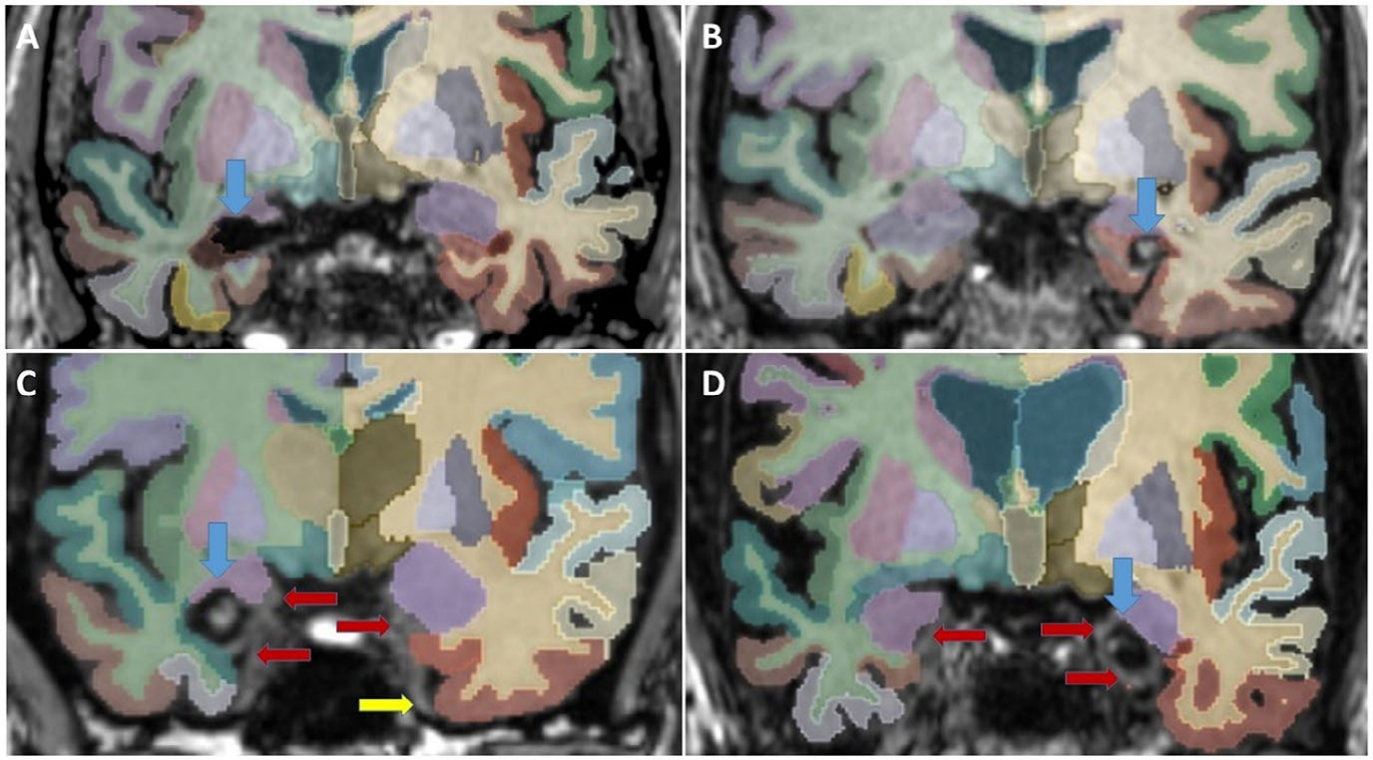
Four coronal slices of post‐surgery segmentations (aparc Desikan–Killiany–Tourville atlas + aseg.deep and orig.mgz). Blue arrows point at the post‐surgery defect. Red arrows point at minimal fail segmentations; the yellow arrow points at a vessel. (A) Right‐sided surgery; no fail segmentations found. (B) Left‐sided surgery hemorrhage in the cavity: no fail segmentations found; (C) right‐sided surgery; and (D) left‐sided surgery, both with minimal fail segmentations of the ipsilateral entorhinal cortex and ipsilateral > contralateral amygdala. The primary olfactory cortex is not segmented in the Desikan–Killiany–Tourville atlas.

Due to a possible influence of asymmetry, we had expected that a missegmentation of the contralateral temporal region might occur after surgery. This was not the case; we only rarely found a minimally missegmented amygdala. We could not detect any failed segmentation of other contralateral volumes, especially not the contralateral entorhinal cortex. Figure 4 demonstrates four post‐surgery segmentations.

Because it is not part of the DKT atlas, we did not segment the primary olfactory cortex and an upper medial area of the amygdala, which consists of the prepiriform cortex and periamygdaloid cortex.

### 3.3 Measured Volumes and Ratings

The mean manually measured postoperative defect size (CSF‐intense on T1‐weighted imaging) was 1427 ± 517 mm^3^ (range 959; 2676 mm^3^; left‐sided mean volume 1411 ± 575 mm; right‐sided mean volume 1453 ± 476 mm^3^).

In a multiparameter correlation matrix, we found a weak and not significant negative correlation between defect size and ILAE outcome (Pearson's correlation coefficient *r* = −0.364; *p* = 0.33).

The mean contralateral ERICA score was 0.23 ± 0.34 pre‐surgery and 0.59 ± 0.58 in the 1‐year follow‐up with a good interrater reliability (ICC [c, 2] = 0.76; 95% confidence interval 0.56–0.87). The mean contralateral Schelten's score was 0.50 ± 0.67 pre‐surgery and 0.64 ± 0.67 in the 1‐year follow‐up with a moderate interrater reliability (ICC [c, 2] = 0.72; 95% confidence interval 0.45–0.87). Both scores did not reveal significant differences (ERICA score *p* = 0.09; Schelten's score *p* = 0.63). Table 3 shows the atrophy ratings.

**TABLE 3:** Mean ratings ± standard deviation of entorhinal cortex atrophy score and mesiotemporal atrophy score.

	Pre‐surgery	1‐year follow‐up
ERICA‐score contralateral	0.23 ± 0.34	0.58 ± 0.59
ERICA‐score contralateral after left‐sided surgery	0.29 ± 0.39	0.79 ± 0.64
ERICA‐score contralateral after right‐sided surgery	0.13 ± 0.25	0.25 ± 0.29
MTA‐score contralateral	0.50 ± 0.67	0.64 ± 0.67
MTA‐score contralateral after left‐sided surgery	0.36 ± 0.48	0.57 ± 0.53
MTA‐score contralateral after right‐sided surgery	0.75 ± 0.96	0.75 ± 0.96

Abbreviations: ERICA‐score, entorhinal cortex atrophy score (0–3); MTA‐score, mesiotemporal atrophy score (0–4).

**FIGURE 5:**
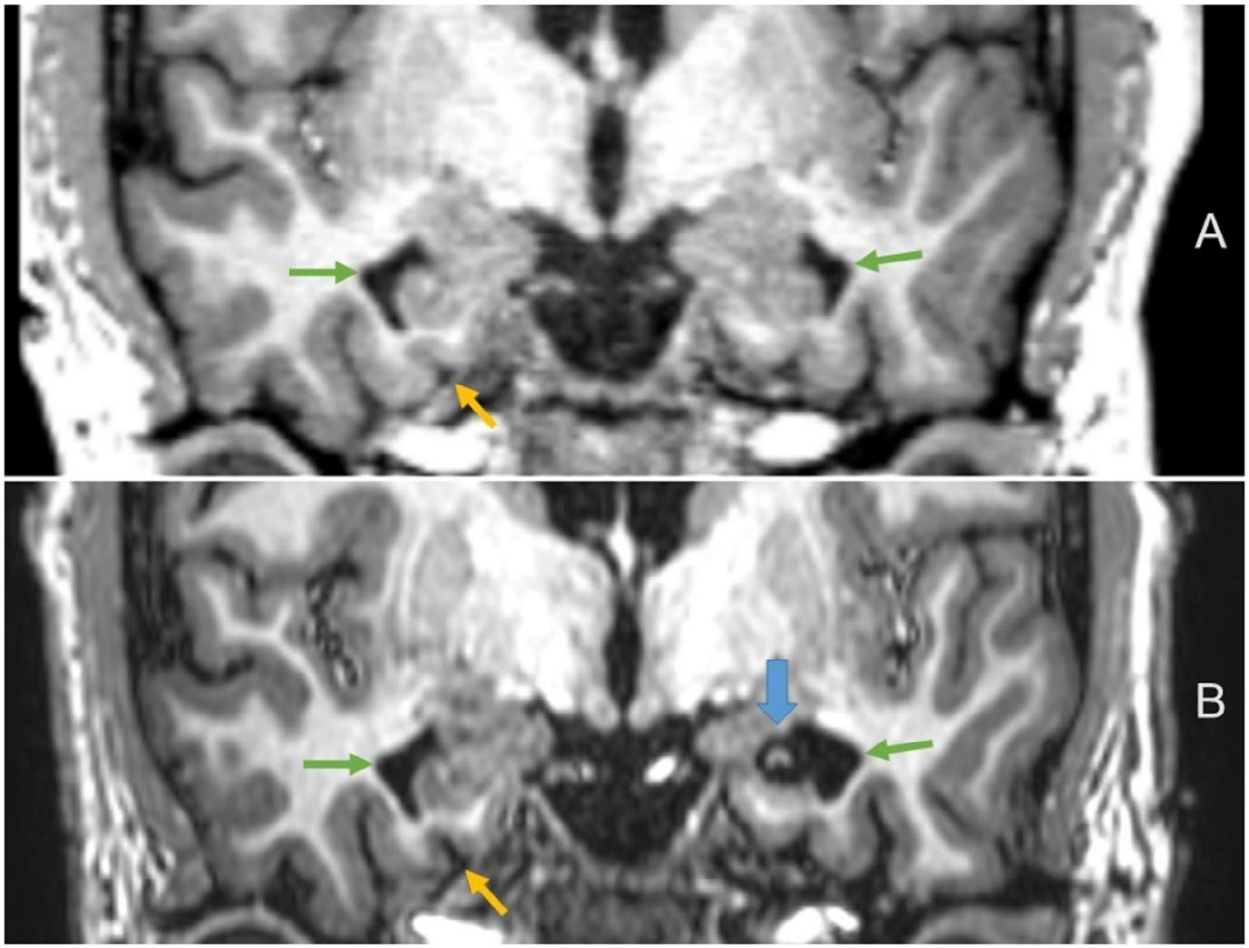
Volume loss of the contralateral entorhinal cortex following laser therapy in a 30‐year‐old‐patient. (A) Pre‐surgery coronal T1‐weighted magnetization prepared—rapid gradient echo; (B) 1‐year follow‐up coronal T1‐weighted Turbo‐Field‐Echo (blue arrow points at the postoperative defect zone after left‐sided surgery; yellow arrows at the widening of the contralateral entorhinal cortex; green arrows at the temporal horns).

However, the ERICA score reveals that we could find the same tendencies in the visual ratings with a contralateral atrophy following left‐sided LITT. Figure 5 demonstrates an example of a widening of the entorhinal cortex in the 1‐year follow‐up.

The additional HypVINN script finished successfully in 4/11 post‐surgery MRIs. But only in one of those four cases was a correct segmentation detected. For this reason, we did not conduct any further statistical analyses on this subscript.

### 3.4 Dominant Hemispheres

We found a right‐sided language dominance in two patients (one of those with left‐sided LITT); in two patients, we found a mixed type (one of those with right‐sided pronunciation as well as left‐sided LITT). In all other patients, a left‐sided language dominance was found.

The exclusion of right‐dominant patients slightly influenced the results, as shown in Table 1. Due to the small sample size, we refrained from further subgroup analyses regarding disease duration, head size, and ILAE seizure freedom outcome at 1 year.

A “mirrored” dependent *t*‐test (*n* = 11) for the contralateral (non‐LITT) side revealed only (not corrected) significant values in the putamen (*p* = 0.003), the accumbens area (*p* = 0.02) as well as the entorhinal and lateral orbitofrontal cortices (*p* = 0.04 for both).

### 3.5 Resting‐State Cortical Parcellation Using Yeo's 17 Networks

The use of Yeo's 17 networks confirmed the results, as detailed in Table 4, demonstrating a right‐hemispheric brain volume reduction following a left‐hemispheric LITT. The highest percentage (mean: 8) of contralateral loss after left‐sided LITT was observed in the right “limbic‐1” area. After right‐sided LITT, no significant atrophy was observed in areas of the left hemisphere.

**TABLE 4:** Mean gray matter volume (mm^3^) of the 17 functional networks from Yeo resting‐state network map and significance *p* without correction.

		Left‐sided LITT and left‐hemispheric language dominance (left, *n* = 5)			Left‐sided LITT (*n* = 7)			Right‐sided LITT (*n* = 4)		
Network name (Yeo17)	Functional network name	Volume pre‐surgery MRI In mm^3^	Volume 1‐year follow‐up MRI in mm^3^	*p*	Volume pre‐surgery MRI in mm^3^	Volume 1‐year follow‐up MRI in mm^3^	*p*	Volume pre‐surgery MRI in mm^3^	Volume 1‐year follow‐up MRI in mm^3^	*p*
Left hemisphere										
17Networks_1	Visual‐1	16,961	16,191	0.067	16,446	15,518	0.010^*^	17,395	17,294	0.878
17Networks_2	Visual‐2	11,103	10,985	0.760	10,686	10,587	0.700	11,474	11,229	0.635
17Networks_3	Motor‐1	16,086	16,387	0.594	15,963	16,132	0.680	17,876	16,989	0.275
17Networks_4	Motor‐2	13,965	14,255	0.326	14,067	14,106	0.880	15,408	15,647	0.703
17Networks_5	Dorsal‐attention‐1	12,797	11,342	0.006^*^	12,506	11,348	0.006^*^	13,287	13,101	0.236
17Networks_6	Dorsal‐attention‐2	10,993	10,568	0.209	10,962	10,545	0.082	12,133	11,939	0.266
17Networks_7	Ventral‐attention	14,065	13,699	0.012^*^	14,125	13,766	0.002^*^	15,210	15,251	0.836
17Networks_8	Frontoparietal‐1	11,160	10,361	0.005^*^	11,044	10,316	0.001^*^	12,152	12,113	0.944
17Networks_9	Limbic‐1	14,480	12,624	0.016^*^	14,033	12,049	0.004^*^	15,245	14,951	0.834
17Networks_10	Limbic‐2	10,487	10,330	0.549	10,373	10,194	0.360	10,839	10,868	0.958
17Networks_11	Frontoparietal‐2	4172	3953	0.180	4129	3944	0.110	4337	4306	0.760
17Networks_12	Frontoparietal‐3	14,729	13,727	0.036^*^	14,643	13,724	0.010^*^	15,329	15,264	0.872
17Networks_13	Frontoparietal‐4	13,335	12,663	0.015^*^	13,256	12,516	0.001^*^	14,666	14,415	0.620
17Networks_14	Motor‐3	7780	7432	0.065	7948	7457	0.011^*^	8299	8273	0.883
17Networks_15	Default‐mode‐network3	5411	4752	0.034^*^	5214	4537	0.007^*^	5309	5292	0.940
17Networks_16	Default‐mode‐network1	20,920	19,646	0.026^*^	20,899	19,711	0.006^*^	22,435	22,704	0.635
17Networks_17	Default‐mode‐network2	30,443	28,782	0.032^*^	30,708	28,947	0.007^*^	34,134	34,222	0.876
Right hemisphere										
17Networks_1	Visual‐1	18,839	18,399	0.270	18,342	17,741	0.126	19,467	18,515	0.130
17Networks_2	Visual‐2	11,213	11,550	0.440	11,136	11,309	0.570	11,862	11,451	0.409
17Networks_3	Motor‐1	15,818	16,330	0.399	16,098	16,473	0.390	18,776	17,720	0.194
17Networks_4	Motor‐2	13,993	13,786	0.048^*^	14,159	13,903	0.045^*^	15,013	14,525	0.163
17Networks_5	Dorsal‐attention‐1	15,577	14,805	0.047^*^	15,215	14,464	0.020^*^	16,267	14,811	0.029^*^
17Networks_6	Dorsal‐attention‐2	10,752	10,732	0.923	10,829	10,743	0.650	12,356	11,922	0.338
17Networks_7	Ventral‐attention	15,979	15,203	0.139	16,041	15,339	0.057	17,299	16,926	0.137
17Networks_8	Frontoparietal‐1	15,241	14,584	0.016^*^	15,287	14,715	0.012^*^	16,213	15,920	0.556
17Networks_9	Limbic‐1	15,520	14,316	0.057	15,585	14,247	0.013^*^	15,338	12,493	0.054
17Networks_10	Limbic‐2	9741	9135	0.032^*^	9591	9159	0.047	9998	9583	0.497
17Networks_11	Frontoparietal‐2	4903	4675	0.287	4861	4666	0.210	5141	5010	0.235
17Networks_12	Frontoparietal‐3	11,696	11,331	0.125	12,001	11,637	0.064	12,644	12,237	0.433
17Networks_13	Frontoparietal‐4	22,129	20,754	0.012^*^	21,578	20,431	0.006^*^	22,716	21,449	0.342
17Networks_14	Motor‐3	9630	9250	0.034^*^	9384	8989	0.004^*^	9767	9056	0.024^*^
17Networks_15	Default‐mode‐network3	4392	4152	0.121	4349	4080	0.020^*^	4568	4385	0.508
17Networks_16	Default‐mode‐network1	22,348	21,570	0.088	21,867	21,177	0.035^*^	24,226	23,594	0.477
17Networks_17	Default‐mode‐network2	14,963	14,508	0.101	14,887	14,348	0.015^*^	15,704	14,426	0.151

Abbreviations: LITT, laser interstitial thermal therapy; mm^3^, cubic millimeter; *n*, number of patients; *p*, probability (significance) value.

* Significant at the corrected significance level of 5%.

### 3.6 Pharmacological Confounding Factors

We did not find any antiepileptic medications containing valproate in the treatment regimens of the included patients, either before or after LITT.

### 3.7 MR Scanners and Sequences

The MRIs on the different scanners were not normally distributed among the patients: Philips (5 pre‐surgery vs. 10, 1‐year follow‐up) versus Siemens (6 pre‐surgery vs. 1, 1‐year follow‐up). We found deviations of up to 5%–10% in the segmented volumes among the different scanners, as already mentioned for FreeSurfer in the literature [26]. The ratio for left versus right TLE for the Siemens scans was 5:1 (pre‐surgery) and 0:1 (1 year follow‐up). The left versus right ratio for the Philips scans was 2:3 (pre‐surgery) and 7:3 (1 year follow‐up).

## 4 Discussion

In our study of 11 patients, we performed a FastSurfer segmentation, visual measurements, and ratings in patients with TLE before and 1‐year after LITT. To the best of our knowledge, no studies to date have addressed whole‐brain volumetry following surgical intervention for TLE. Technically, automated volumetry—typically optimized for “complete and healthy” brains—also poses additional challenges. After minimally invasive surgery, FastSurfer enabled volumetric analysis for each patient in our group. However, it remains uncertain whether this approach can be applied uniformly to all patients following other approaches, for example, temporal lobe resection.

We found significant right‐hemispheric volume losses of the thalamus and temporal structures, for example, the fusiform cortex, after left‐sided surgery. This could be explained by a left‐brain dominated coupling through the Papez circuit [27]. The additional application of Yeo's functional parcellation confirmed the results. A major limitation of this study is that the MRI scans were conducted on two different devices, which may have introduced variability and influenced the results.

LITT [6] is the minimal invasive alternative for anterior temporal lobe resection [28] or selective amygdalo‐hippocampectomy [7] in refractory TLE. Nonetheless, minimal‐invasive surgery could influence the functional and neurocognitive status of the patients [29]. Seizure recurrence has occurred with all of these surgical approaches [30]. The extent of brain surgery significantly affects the long‐term impact on remote brain structures [31].

The overall outcome of LITT was not associated with the ablation volume in a meta‐analysis, in which the mean ablation volume was 5376 mm^3^, and seizure freedom was achieved in 58% of TLE cases [32]. In comparison, our study reported a mean ablation volume of only 1427 mm^3^ and a seizure freedom rate of 55%. Some studies suggest that a resection or ablation of the piriform cortex can positively influence the outcome [33].

There is still a lot to learn with this new technique, and the ablated volumes also seem to vary greatly, depending on the patient [34]. The use of adaptive neuronal nets and/or deep learning algorithms, such as FastSurfer, already works successfully in many (mostly neurodegenerative) diseases [35, 36]. We could demonstrate that it can also be used successfully in postoperative patients after LITT. The use of volumetric software after surgery is generally promising but must be adapted accordingly to the size of defect, for example, with deep learning algorithms for resection cavities [37]. In the context of minimally invasive techniques and long‐term MRI monitoring, the question always arises as to where the defect ends and where an ex vacuo dilation of the ventricular system begins. In some cases, this distinction may not be conclusively resolved.

Major limitation of our study is the small patient number. Due to the high number of variables tested, no significant statement can be made. Another limitation is the use of two different MR scanners. Theoretically, the differences measured in the temporal lobes on both sides could also be due to the inter‐scanner deviations [26]. However, this is contradicted by the fact that the temporal horn diameter has also changed on the contralateral side. We were also able to confirm these trends in the visual ratings.

In addition, not all patients were free of symptoms after surgery, so the measured atrophy could also be a result of the ongoing disease or continued medication [38].

The volumetry of surgically treated brains presents a challenge for most software solutions. This could lead to the distortion of the results; therefore, we verified all segmentations visually.

The size of our visually measured defect zones was smaller than in the previously published literature. One reason for this could be that the visual measurements were conducted solely on the CSF defect in the 1‐year follow‐up T1‐weighted data and may not accurately represent the complete defect size.

The observation period was relatively short, set at 1 year, to detect significant effects. Future studies should include 2‐ or 5‐year follow‐up assessments.

The long‐term impact of minimally invasive procedures in epilepsy surgery is becoming increasingly significant, highlighting the importance of future research into their mechanisms and long‐term effects [39]. The harmonization and standardization of MRI techniques, for example, T1‐mappings [40], and measurement methods can contribute to improved patient monitoring.

Our study indicates that undetected effects of minimally invasive epilepsy surgery for TLE exist. A similar atrophy mechanism to that observed in strokes is hypothesized. In addition to the ipsilateral atrophy of brain structures, these could also include contralateral regions included in the Papez circuit.

## Ethics Statement

We confirm that we have read the Journal's position on issues involved in ethical publication and affirm that this report is consistent with those guidelines.

## Conflicts of Interest

The authors declare no conflicts of interest.
